# Three-dimensional Weyl topology in one-dimensional photonic structures

**DOI:** 10.1038/s41377-022-00886-6

**Published:** 2022-06-17

**Authors:** Kosmas L. Tsakmakidis, Tomasz P. Stefański

**Affiliations:** 1 Section of Condensed Matter Physics, Department of Physics, National and Kapodistrian University of Athens, Panepistimioupolis, GR-157 84 Athens, Greece; 2 Faculty of Electronics, Telecommunications, and Informatics, Gdansk University of Technology, 80-233 Gdansk, Poland

**Keywords:** Photonic crystals, Metamaterials

## Abstract

Topological features, in particular distinct band intersections known as nodal rings, usually requiring three-dimensional structures, have now been demonstrated experimentally in an elegantly simple one-dimensional photonic crystal.

One of the most fascinating advances in modern optics and photonics has been the emergence and development—during approximately the past decade^1^—of ‘topological’ structures, where the presence or not of surface waves depends on the precise energy-momentum structure of bulk bands, thereby allowing for robust propagation of the surface states, largely immune to defects, fabrication imprecisions, disorder and roughness^1^. Particular attention has been directed towards, so called, Weyl points^2^—single-point band-degeneracies with a linear dispersion in three-dimensional momentum space—and ‘nodal lines’ formed at the intersections of energy bands^3^. The reasons are, first, because of their inherent interest from a topological-classification point of view, and second because Weyl points are stable to perturbations when one of the inversion (P) or time-reversal (T) symmetries is broken. Nodal lines, on the other hand, feature unique two-dimensional surface waves, known as drumhead edge states, and exhibit unusual non-Abelian band topology. Moreover, being one dimension higher than Weyl points, these line loops in the momentum space (see Fig. 1a) can exhibit a variety of shapes, e.g., nodal knots or Hopf links, as well as nodal chains for multiple nodal lines^4^.

**Fig. 1:**
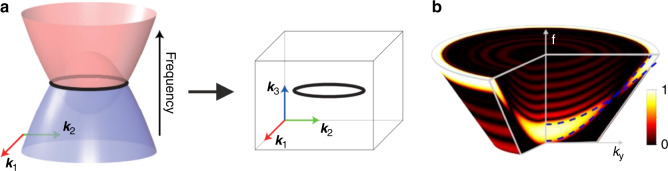
Topological Weyl nodal loops. **a** When two energy (or frequency) - momentum bands intersect, that is, when they are degenerate (same energy but different mode profiles), they can form a closed loop at the points where they touch, also known as a nodal ring. **b** Angle-resolved reflection spectra of one of the 1D photonic structures of Ref. ^5^, showing two upwards-curvy bands (highlighted with dashed blue lines) touching, thereby forming a circular ring in the 3D energy-momentum space

Despite these ongoing exciting theoretical developments and understandings, the fabrication of the underlying photonic structures has typically been challenging, hindering their large-scale usage and exploitation for practical devices. For instance, so far, these three-dimensional (3D) structures involved special double gyroids constructions or other intricate 3D photonic or phononic crystals, which, while suitable for fundamental and proof-of-concept studies, are not fitting for many real-world applications—particularly at visible wavelengths and for integrated nano-photonic functions. Now, writing on p. 134 of the 11th issue of *Light: Science & Applications*, Wei-Min Deng and Jian-Wen Dong along with colleagues, report^5^ the conception and experimental demonstration of *ideal* (exactly circular) nodal rings in simple, one-dimensional photonic crystals in the visible regime, alleviating a significant obstacle in the potential wider deployment of Weyl topological features in optoelectronic devices.

The starting point in the authors’ analysis is a plain 1D photonic crystal, with a unit cell made of only two dielectrics layered in the *z* direction (with a total height *a*), and then being periodically repeated on the *xy* plane. The Brillouin zone resulting from such a structure is that of a slab constrained by two planes at *k*_*z*_ =± π/*a*. Here, the authors considered the in-plane wavevector-*k* components (usually disregarded in the analyses of 1D structures), and noticed that with increasing *k*_*y*_ the energy bands of the structure split and start bending upwards with different slopes—implying that they eventually cross at some point (*k*_*y*_ value). Crucially, since this is only a 1D photonic crystal, the band diagram is symmetrical along the *k*_*x*_ and *k*_*y*_ directions, thereby giving rise to a rotationally symmetric series of ‘Weyl points’, that is, to a circular loop formed by the intersection of two bands, also known as an ideal gapless (no gap between the bands) nodal ring. A tell-tale signature of the topological nature of this ring is that it exhibits a ‘topological charge’ of π—where by a topological charge one refers to a special topological *invariant*; a conserved quantity (in the absence of topological phase transitions) characterizing the topological phase of the structure, making it robust against structural perturbations. These topological invariants are, thus, often called ‘topological charges’ similarly to electric or magnetic charges of electric or magnetic monopoles respectively. And the exhibited value of π for such a charge, measured experimentally in Ref. ^5^ with angle-resolved reflectometry (a standard technique), unmistakably shows that we are indeed dealing with a *topological* nodal ring (see Fig. 1b).

Further, the authors of Ref. ^5^ did not stop there but also demonstrated the existence of unique surface states at the boundary of two 1D photonic crystals with slightly different optogeometric parameters. Because each 1D photonic crystal is topological, one may predict, by studying only its bulk properties (modes), that each one supports a wave on its surface—thereby, merging the crystals together, we expect two such edge states to exist at the crystals’ common interface. This is indeed what the authors observe in their measurements based again on angle-resolved reflection spectra, where a dip in the attained spectra is a direct indication of the excitation of a wave on the structure. Two such dips are experimentally clearly seen in the expected spectral region, thereby corroborating their theoretical predictions.

The simple route that Wei-Min Deng et al. provide for attaining 3D topological properties in 1D periodic structures is yet another example of ‘extreme’ light behavior engineered in suitably designed photonic crystal or metamaterial structures^6^, and may find a whole host of applications, from on-chip optical isolators^7^ and topological lasing^8^ to slow and stopped light^9^, and from nonlinear to quantum optics^10^—more generally, for applications requiring strong light-matter interactions^11^. But for now, the elegantly simple path that Ref. ^5^ identified for exploiting and harnessing 3D topological features in 1D structures helps to remind one the well-known excerpt that ‘*all things are difficult before they are easy*.’
